# A dominant function of CCaMK in intracellular accommodation of bacterial and fungal endosymbionts

**DOI:** 10.1111/j.1365-313X.2010.04228.x

**Published:** 2010-05-11

**Authors:** Teruyuki Hayashi, Mari Banba, Yoshikazu Shimoda, Hiroshi Kouchi, Makoto Hayashi, Haruko Imaizumi-Anraku

**Affiliations:** National Institute of Agrobiological SciencesTsukuba, Ibaraki 305–8602, Japan

**Keywords:** arbuscular mycorrhizal symbiosis, CCaMK, common symbiosis genes, intracellular infection, root nodule symbiosis

## Abstract

In legumes, Ca^2+^/calmodulin-dependent protein kinase (CCaMK) is a component of the common symbiosis genes that are required for both root nodule (RN) and arbuscular mycorrhiza (AM) symbioses and is thought to be a decoder of Ca^2+^ spiking, one of the earliest cellular responses to microbial signals. A gain-of-function mutation of CCaMK has been shown to induce spontaneous nodulation without rhizobia, but the significance of CCaMK activation in bacterial and/or fungal infection processes is not fully understood. Here we show that a gain-of-function CCaMK^T265D^ suppresses loss-of-function mutations of common symbiosis genes required for the generation of Ca^2+^ spiking, not only for nodule organogenesis but also for successful infection of rhizobia and AM fungi, demonstrating that the common symbiosis genes upstream of Ca^2+^ spiking are required solely to activate CCaMK. In RN symbiosis, however, CCaMK^T265D^ induced nodule organogenesis, but not rhizobial infection, on Nod factor receptor (NFRs) mutants. We propose a model of symbiotic signaling in host legume plants, in which CCaMK plays a key role in the coordinated induction of infection thread formation and nodule organogenesis.

## Introduction

Recent studies have revealed the host legume genes that regulate RN and/or AM symbioses (Oldroyd and Downie, 2008; Parniske, 2008). RN symbiosis begins with the specific recognition of rhizobial Nod factors (NFs) (Ardourel *et al.*, 1994; Lerouge *et al.*, 1990; López-Lara *et al.*, 1995; Niwa *et al.*, 2001) by LysM receptor kinases of the compatible host plants. In *Lotus japonicus*, two LysM receptor kinases, NFR1 and NFR5 (Madsen *et al.*, 2003; Radutoiu *et al.*, 2003, 2007), are essential for perception of Nod factors secreted from *Mesorhizobium loti* (López-Lara *et al.*, 1995; Niwa *et al.*, 2001). This compatible recognition induces intracellular Ca^2+^ signals, i.e. Ca^2+^ influx at the tip of root hairs followed by Ca^2+^ spiking, an oscillation of cytosolic Ca^2+^ concentration around the peri-nuclear region of root hair cells (Ehrhardt *et al.*, 1996; Miwa *et al.*, 2006; Shaw and Long, 2003). Genetic and molecular studies have positioned NFR1 and NFR5 upstream of both Ca^2+^ signals, because either *nfr1* or *nfr5* mutants were defective in the generation of both Ca^2+^ influx and Ca^2+^ spiking upon Nod factor application. On the other hand, NIN (Marsh *et al.*, 2007; Schauser *et al.*, 1999), NSP1 and NSP2 (Heckmann *et al.*, 2006; Kaló*et al.*, 2005; Murakami *et al.*, 2006; Smit *et al.*, 2005), which are putative transcription factors, function downstream of both Ca^2+^ signals (Miwa *et al.*, 2006). NSP1, NSP2 and NIN have been shown to be necessary for nodule organogenesis and rhizobial infection, which is accompanied by formation of infection threads (ITs). NFR1, NFR5, NSP1, NSP2 and NIN are only required for RN symbiosis, but not for AM symbiosis.

Among the genes required for both RN and AM symbioses (i.e. common symbiosis genes), *SYMRK* (Endre *et al.*, 2002; Stracke *et al.*, 2002), *CASTOR* and *POLLUX* (Ané*et al.*, 2004; Imaizumi-Anraku *et al.*, 2005), *NUP85* (Saito *et al.*, 2007) and *NUP133* (Kanamori *et al.*, 2006) are positioned upstream of Ca^2+^ spiking (Miwa *et al.*, 2006) and believed to be required for generation of Ca^2+^ spiking in response to the infection signals released from symbiotic partners. However, mutations in these ‘upstream genes’ do not affect the elicitation of Ca^2+^ influx in response to Nod factors. The other common symbiosis gene, *CCaMK* (Gleason *et al.*, 2006; Lévy *et al.*, 2004; Tirichine *et al.*, 2006) and *CYCLOPS* (Yano *et al.*, 2008) lie downstream of Ca^2+^ spiking (Miwa *et al.*, 2006) and act together as a signal transduction complex required for infection (Yano *et al.*, 2008). CCaMK is a strong candidate for the decoder of Ca^2+^ spiking, on the basis of its domain structure, which is composed of a serine/threonine kinase domain, a calmodulin (CaM) binding domain and three EF-hand motifs that potentially trap Ca^2+^ ions (Lévy *et al.*, 2004; Yang *et al.*, 2007). In *L. japonicus*, a gain-of-function CCaMK mutant *snf1*, in which Thr at the autophosphorylation site of the kinase domain was substituted by Ile, developed spontaneous nodules in the absence of rhizobia (Tirichine *et al.*, 2006). In addition, point or truncated mutations of CCaMK, which lead to loss of auto-inhibition, resulted in the formation of spontaneous nodules in *Medicago truncatula* (Gleason *et al.*, 2006). These results indicate that activation of CCaMK is necessary and is also sufficient for nodule organogenesis (Gleason *et al.*, 2006; Tirichine *et al.*, 2006). Besides CCaMK, a gain-of-function LHK1 (*Lotus* histidine kinase 1) has been identified from the *snf2* mutant, which also shows spontaneous nodulation (Tirichine *et al.*, 2007). LHK1 encodes a cytokinin (CK) receptor kinase and substitution of Leu266 by Phe in the receptor domain confers CK-independent activity (Tirichine *et al.*, 2007). Together with *hit1*, a loss-of-function mutant of LHK1 (Murray *et al.*, 2007), these mutants indicate the involvement of CK signaling in nodule organogenesis.

In the RN symbiosis, coordinated regulation between rhizobial infection and nodule organogenesis is essential for the development of fully effective nodules (Frugier *et al.*, 2008; Oldroyd and Downie, 2008). Since almost all symbiotic genes described above have been isolated from loss-of-function mutants (Crespi and Frugier, 2008), i.e. non-nodulating mutants, the roles of individual symbiotic genes in infection and/or nodule organogenesis processes remain elusive. Hitherto, a number of schemes have been proposed to explain the mechanism underlying the guidance and control system for rhizobial infection. These models were devised on the basis of symbiotic defects in nodulating mutants, i.e. loss-of-function of Ca^2+^ spiking, Ca^2+^ influx, root hair deformation, IT formation, cortical cell division and gene expression upon rhizobial infection or NF treatment (Ardourel *et al.*, 1994; Geurts *et al.*, 2005; Marsh *et al.*, 2007; Miwa *et al.*, 2006; Murray *et al.*, 2007; Smit *et al.*, 2007; Tirichine *et al.*, 2007; Wais *et al.*, 2000; Yano *et al.*, 2008). Recent identification of gain-of-function mutants and of its causative genes prompted us to examine the epistatic relationships between symbiotic genes. However, recent models have focused on the regulation pathways for nodule organogenesis (Gleason *et al.*, 2006; Marsh *et al.*, 2007; Tirichine *et al.*, 2006, 2007; Yano *et al.*, 2008) and it remains unclear whether those symbiotic genes are involved in infection processes directly or indirectly. To understand the function of symbiotic genes in bacterial and/or fungal intracellular symbiotic processes, we examined the phenotypes of a diverse array of symbiotic gene mutants after transformation with a gain-of-function CCaMK (CCaMK^T265D^) (Gleason *et al.*, 2006; Rasmussen and Rasmussen, 1994; Sheen, 1996; Waldmann *et al.*, 1990), for nodule organogenesis and rhizobial and/or mycorrhizal infection. We also evaluated the epistatic interactions between a gain-of-function LHK1 (LHK1^L266F^) and the other symbiotic genes on the basis of their nodulation phenotypes. Our results indicate that activation of CCaMK through upstream genes is prerequisite to allow infection of rhizobia and AM fungi. Furthermore, intracellular infection of rhizobia through ITs requires another signaling pathway derived from NFR1 and NFR5 besides the pathway involving Ca^2+^ spiking mediated by common symbiosis genes. We show here the crucial roles of CCaMK in intracellular symbioses.

## Results

### CCaMK^T265D^ induces spontaneous nodulation and fully complements CCaMK loss-of-function mutants

**Figure 1:**
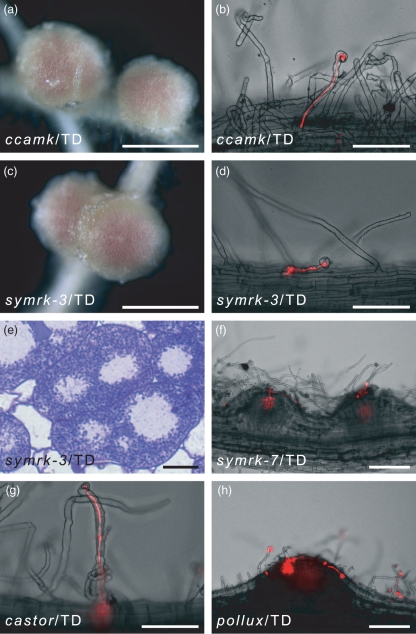
Complementation of rhizobial infection phenotypes of non-nodulating mutants by CCaMK^T265D^ transformation. (a–h) Transformed plants were inoculated with DsRed-labeled *Mesorhizobium loti.* (a, c) Mature nodules formed on the roots of *ccamk-3*/CCaMK^T265D^ (*ccamk*/TD) and *symrk-3*/CCaMK^T265D^ (*symrk-3*/TD) after 4 weeks of inoculation. Scale bars are 1 mm. (b, d, g) Root hairs of *ccamk*/TD, *symrk-3*/TD and *castor-4*/CCaMK^T265D^ (*castor*/TD) 2 weeks after inoculation, shown as merged images of bright-field and fluorescence images (DsRed). Infection threads can be seen inside the curled root hairs. Scale bars are 100 μm. (e) A mature nodule section of *symrk-3*/TD stained with toluidine blue. The nodule was filled with differentiated bacteroids. Scale bar is 20 μm. (f, h) Nodule primordia with rhizobial infection on the roots of *symrk-7*/CCaMK^T265D^ (*symrk-7*/TD) and *pollux-2*/CCaMK^T265D^ (*pollux*/TD), shown as merged images of bright-field and fluorescence images (DsRed). Scale bars are 200 μm.

**Figure 2:**
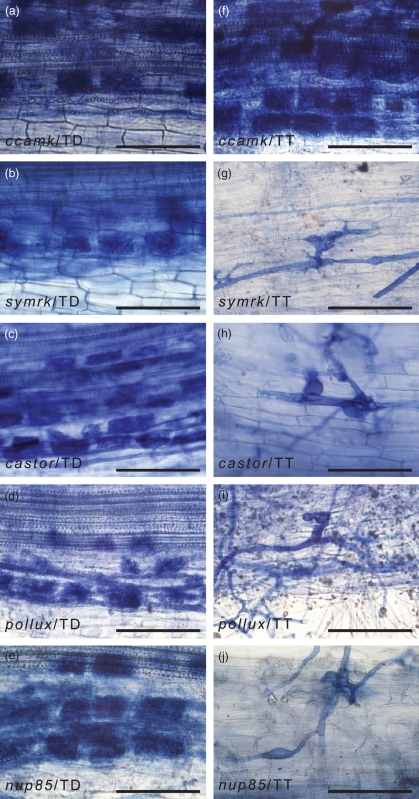
Complementation of mycorrhization phenotypes of *ccamk-3* and upstream mutants by CCaMK^T265D^ or wt-CCaMK transformation. (a–j) Symbiotic phenotypes of transformed plants were observed 4 weeks after inoculation with *Glomus intraradices.* (a–f) Roots of *ccamk-3*/CCaMK^T265D^ (*ccamk*/TD), *symrk-7*/CCaMK^T265D^ (*symrk*/TD), *castor-4*/CCaMK^T265D^ (*castor*/TD), *pollux-2*/CCaMK^T265D^ (*pollux*/TD) and *nup85-3*/CCaMK^T265D^ (*nup85*/TD) as well as *ccamk-3*/wt-CCaMK (*ccamk*/TT) were filled with well developed arbuscules. (g–j) In the case of *symrk-7*/wt-CCaMK (*symrk*/TT), *castor-4*/wt-CCaMK (*castor*/TT), *pollux-2*/wt-CCaMK (*pollux*/TT) and *nup85-3*/wt-CCaMK (*nup85*/TT), mycorrhizal invasion was aborted in the epidermis and only running hyphae (g) and swollen appressoria (h, i) were observed. All scale bars are 100 μm.

It has been reported that substitution of Thr at the autophosphorylation site in the kinase domain by Asp confers Ca^2+^ independent activation of CCaMKs and CaMKII (Gleason *et al.*, 2006; Rasmussen and Rasmussen, 1994; Sheen, 1996; Waldmann *et al.*, 1990). To evaluate the efficiency of CCaMK^T265D^ in which Thr265 was substituted by Asp, it was expressed in the *Lotus ccamk-3* mutant under the control of the CaMV 35S promoter by hairy root transformation. The transformed roots showed spontaneous nodulation under mock inoculation (Table 1; Figure S1c,d). To avoid the possibility that ectopic expression of CCaMK led to spontaneous nodulation, CaMV35S-CCaMK^T265T^ (wild type CCaMK, denoted as wt-CCaMK hereafter) was also transformed into *ccamk-3.* No spontaneous nodulation was induced by ectopic wt-CCaMK expression (Table 1; Figure S1e,f). Furthermore, rhizobial and mycorrhizal infections were restored by CCaMK^T265D^ as well as by wt-CCaMK transformation (Table 1; Figures 1a, 2a,f and S1a,b), indicating that CCaMK^T265D^ is fully functional in the infection processes in *Lotus* roots.

**Table 1:** Induction of spontaneous nodulation and restoration of symbiotic defective phenotypes of non-nodulating mutants, transformed with wt-CCaMK (TT) or CCaMK^T265D^ (TD) constructs

		Phenotypes		
*Lotus* lines	CCaMK construct	SpN^a^	Nod^b^	AM^c^
Gifu (B-129)	TT	0/24	22/23	33/33
Gifu (B-129)	TD	29/43	21/21	25/25
*ccamk-3*	TT	0/19	31/32	24/26
*ccamk-3*	TD	57/77	32/35	21/27
*nfr1-4*	TT	0/91	0/29	nt
*nfr1-4*	TD	33/93	19/38^d^	nt
*nfr5-2*	TT	0/27	0/30	nt
*nfr5-2*	TD	22/32	19/33^d^	nt
*symrk-3*	TT	0/5	0/20	nt
*symrk-3*	TD	12/18	27/42	nt
*symrk-7*	TT	0/36	0/40	0/24
*symrk-7*	TD	23/54	37/41	37/38
*castor-4*	TT	0/51	0/54	0/19
*castor-4*	TD	51/67	58/67	19/25
*pollux-2*	TT	0/58	0/46	0/16
*pollux-2*	TD	44/66	46/52	14/17
*nup85-3*	TT	0/38	0/41	0/20
*nup85-3*	TD	25/26	41/46	28/31
*cyclops-4*	TT	–e	15/15^f^	0/9
*cyclops-4*	TD	–e	23/27^f^	4/17^g^
*nsp2-1*	TT	0/72	0/51	nt
*nsp2-1*	TD	0/96	0/59	nt
*nin-2*	TT	0/25	0/32	nt
*nin-2*	TD	0/36	0/30	nt
*hit1-1*	TT	0/64	6/22^h^	nt
*hit1-1*	TD	0/73	9/50^h^	nt

a Spontaneous nodulation in the absence of *Mesorhizobium loti* (scored at 6 weeks after transplantation).

b Nodule formation under *M. loti* inoculation.

c Mycorrhization with arbuscule formation.

d Empty nodule without rhizobial invasion.

e Previously reported by Yano *et al.* (2008).

f Bump-like structure without rhizobial invasion.

g Formation of few arbuscules.

h Infected nodule with abnormal shape.

nt, not tested.

Number of plants with the phenotypes describe above (a–h) per number of transformed plants are listed. Infection phenotypes were examined 4 weeks after inoculation. Data were compiled from more than two independent experiments.

### CCaMK^T265D^ dispenses the requirement of ‘upstream genes’ for not only nodule organogenesis but also for rhizobial infection through ITs

We examined the function of CCaMK in mutants that are defective in common symbiosis genes, viz: *SYMRK*, *CASTOR*, *POLLUX* and *NUP85.* As these common symbiosis genes have been shown to be required for the generation of Ca^2+^ spiking, they are supposed to function upstream of CCaMK and thus are denoted as ‘upstream genes.’ Under mock inoculation, expression of CCaMK^T265D^ resulted in formation of spontaneous nodules on the roots of the upstream gene mutants (Table 1; Figure S2c–f), indicating that CCaMK^T265D^ could obviate the requirement of the upstream genes for nodule organogenesis.

The upstream gene mutants transformed with CCaMK^T265D^ formed fully mature and functional nodules upon *M. loti* inoculation (Figure 1). Using DsRed-labelled *M. loti* (Maekawa *et al.*, 2009), we found occurrence of successful infection events comparable to those in wild type plants, including root hair curling with micro-colonies, ITs that were developed within curled root hairs and ramified infection thread networks towards the central zone of nodules (Figure 1b,d,f–h). Among upstream genes, *SYMRK* has been implicated to be involved in infection processes of rhizobia (Bersoult *et al.*, 2005; Capoen *et al.*, 2005; Limpens *et al.*, 2005). *SYMRK* encodes protein kinase with leucine-rich repeat and its non-legume orthologs have been proved to be functional in RN and/or AM symbioses (Markmann *et al.*, 2008). In *M. truncatula*, knockdown or ectopic expression of DMI2, a *Medicago* ortholog of SYMRK, resulted in the development of aberrant ITs within nodules (Capoen *et al.*, 2005; Limpens *et al.*, 2005). Analyses using DMI2-GFP fusion revealed that DMI2 is localized on the plasma membrane and infection thread membrane at the distal part of the infection zone, suggesting the involvement of DMI2 in symbiosome formation (Capoen *et al.*, 2005; Limpens *et al.*, 2005). In addition, the fact that MtHMGR1 (Kevei *et al.*, 2007) and SIP1 (Zhu *et al.*, 2008) interact with the kinase domain of SYMRK, suggests that a signaling pathway(s) other than the one mediated by common symbiosis genes is crucial for rhizobial infection (Holsters, 2008). To exclude the possibility of residual activity of *symrk-7*, which retains most of the kinase domain of SYMRK (Kistner *et al.*, 2005; Stracke *et al.*, 2002), we examined the phenotypes of *symrk-3*, which lacks the kinase domain completely (Kistner *et al.*, 2005; Stracke *et al.*, 2002), when transformed with CCaMK^T265D^. Spherical, pink nodules with differentiated bacteroids were formed on the roots of *symrk-3*/CCaMK^T265D^ (Figure 1c,e), as well as on those of *symrk-7*/CCaMK^T265D^ (Figure 1f), similar to wild type nodules. On those roots, neither white nodules nor nodules with aberrantly developed infection threads were observed. These results indicate that SYMRK is required solely for activation of CCaMK in rhizobial infection processes, as well as other upstream genes.

### Upstream genes are only required for the activation of CCaMK in both rhizobial and mycorrhizal infection processes

Besides overcoming rhizobial infection defects, CCaMK^T265D^ could also complement defects in mycorrhizal infection in all upstream mutants examined (Table 1; Figure 2). Mutant roots expressing CCaMK^T265D^ were filled with well-developed arbuscules (Figure 2b–e), while no endosymbiotic structures were observed in the mutants/wt-CCaMK (Figure 2g–j). In *symrk-7*/wt-CCaMK roots, we found only running hyphae on the root surface (Figure 2g). Abnormally-shaped appressoria were formed on the roots of *castor-4*/wt-CCaMK, *pollux-2*/wt-CCaMK and *nup85-3*/wt-CCaMK (Figure 2h–j), indicating that wt-CCaMK did not suppress the epidermal block for mycorrhizal invasion in these mutants. These results suggest that CCaMK^T265D^ could function in a similar way to wt-CCaMK activated by infection signals from rhizobia or mycorrhizae through the upstream genes and strengthen the idea that upstream genes are only required for the activation of CCaMK in rhizobial and mycorrhizal infection processes.

### Rhizobial and mycorrhizal infection processes are CYCLOPS-dependent

**Figure 3:**
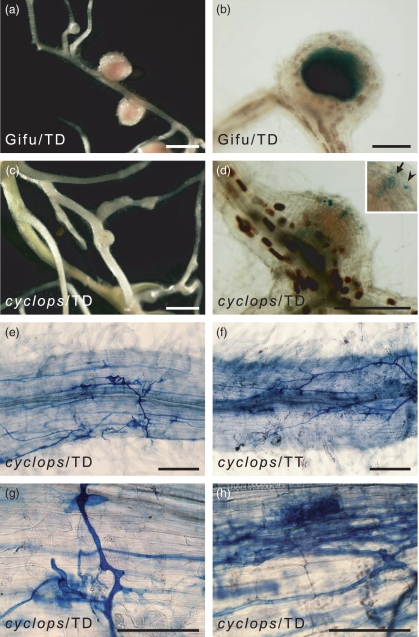
Complementation of rhizobial infection phenotypes and mycorrhization phenotypes of *cyclops* mutant by CCaMK^T265D^ or wt-CCaMK transformation. Symbiotic phenotypes of transformed plants were observed 4 weeks after inoculation with lacZ-labelled *Mesorhizobium loti* (a–d) or *Glomus intraradices* (e–h). (a, c) Mature nodules on the roots of wild-type/CCaMK^T265D^ (Gifu/TD) and bump-like structures on the roots of *cyclops-4*/CCaMK^T265D^ (*cyclops*/TD) were formed. Scale bars are 1 mm. (b, d) Rhizobial infection was confirmed by lacZ staining. Effective nodules with rhizobial infection were formed on the roots of Gifu/TD (b), but rhizobial infection was aborted at the epidermis on bump-like structures on the roots of *cyclops*/TD (d). The inset shows magnified view of the aborted infection thread (arrow) and the micro-colony (arrowhead). Scale bars are 500 μm. (e–h) Roots of *cyclops*/TD were filled with arbuscules only occasionally (h), fungal invasion was aborted in the epidermis and only running hyphae (e) and swollen appressoria (g) were observed in the roots of *cyclops-4*/TD, as well as *cyclops-4*/wt-CCaMK (f). Scale bars are 200 μm (e,f) and 100 μm (g, h).

Among the common symbiosis genes identified so far, *CYCLOPS* is positioned downstream of Ca^2+^ spiking (Miwa *et al.*, 2006). The *cyclops* mutants abort intracellular infection by rhizobia and AM fungi. IT development accompanied by rhizobial infection was arrested within curled root hairs, leading to formation of small bumps with no bacteria inside. For the AM symbiosis, hyphal penetration through the epidermis was blocked, although arbuscules were formed at a very low frequency (Kistner *et al.*, 2005; Yano *et al.*, 2006, 2008). At the molecular level, CYCLOPS has been shown to interact with CCaMK *in planta* and be phosphorylated by CCaMK *in vitro*, suggesting that CYCLOPS acts in concert with CCaMK to regulate intracellular symbioses (Yano *et al.*, 2008). Yano *et al.* (2008) also reported that nodule organogenesis is independent of CYCLOPS, because spontaneous nodules were formed in *cyclops-4*/CCaMK^T265D^ roots under mock inoculation. To examine whether CYCLOPS is involved in infection processes, CCaMK^T265D^ was transformed into the *cyclops-4* mutant. CCaMK^T265D^, as well as wt-CCaMK, did not restore rhizobial infection defects of *cyclops-4* (Table 1). On the roots of *cyclops-4*/CCaMK^T265D^, bump formation was observed in which rhizobial invasion was aborted within root hairs (Figure 3c,d). In the case of AM symbiosis, hyphal penetration was aborted in epidermis or outer cortical cell layers, except for rare occasions where a few internal hyphae and/or arbuscules developed on the roots of *cyclops-4*/CCaMK^T265D^ (Table 1; Figure 3e,g,h). These results are in contrast to those for upstream mutants/CCaMK^T265D^ roots in which the cortical cell layer was filled with numerous arbuscules (Figure 2a–e). We conclude that CYCLOPS is epistatic to CCaMK^T265D^ in respect to rhizobial and mycorrhizal infection processes, opposite to the case of nodule organogenesis.

### NFR1 and NFR5 are indispensable for rhizobial infection through root hair ITs

**Figure 4:**
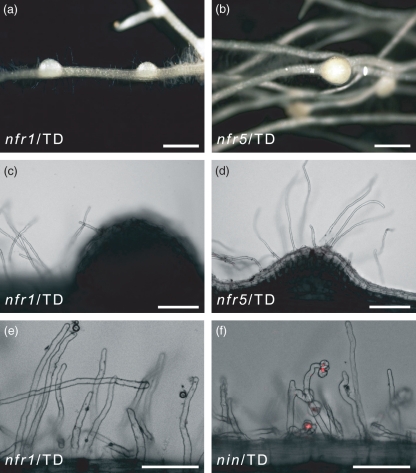
Complementation of rhizobial infection phenotypes of *nfr1* and *nfr5* mutants by CCaMK^T265D^ transformation. (a–f) Transformed plants were inoculated with DsRed-labelled *M. loti.* (a, b) The empty nodules formed on the roots of *nfr1-4*/CCaMK^T265D^ (*nfr1*/TD) and *nfr5-2*/CCaMK^T265D^ (*nfr5*/TD) after 4 weeks of inoculation. Scale bars are 1 mm. (c–f) Bright-field and fluorescence (DsRed) images were merged into single images. (c, d) Nodule primordia without rhizobial infection on the roots of *nfr1*/TD and *nfr5*/TD. Scale bars are 200 μm. (e, f) Root hairs of *nfr1*/TD and *nin-2*/CCaMK^T265D^ (*nin*/TD) 2 weeks after inoculation. Scale bars are 100 μm. (e) Neither bacterial colonization nor infection thread formation was observed on the roots of *nfr1*/TD. (f) Aberrant curled root hairs with micro-colonies were observed on the roots of *nin*/TD.

NFR1 and NFR5, putative NF receptors, are considered to be the starting point of the RN symbiosis in *Lotus.* Indeed, their corresponding mutants lack any symbiotic responses, including Ca^2+^ signals in response to *M. loti* NFs (Miwa *et al.*, 2006). Introduction of CCaMK^T265D^ in the *nfr1-4* or *nfr5-2* mutants resulted in spontaneous nodulation under mock inoculation (Table 1; Figure S2a,b). However, in contrast to the mutants of upstream genes described above, although nodule-like structures developed, neither bacterial colonization nor root hair ITs were found on the roots of both *nfr1-4*/CCaMK^T265D^ and *nfr5-2*/CCaMK^T265D^ upon inoculation of *M. loti* (Table 1; Figure 4a–e). In *nfr1-4*/CCaMK^T265D^ roots, no root hair deformation occurred (Figure 4c,e), as well as in *nfr5-2*/CCaMK^T265D^ roots. These results indicate that NFR1 and NFR5, upon perception of Nod factors, may generate a signal or signals other than the one mediated by the common symbiosis genes and that these signals are required for infection of rhizobia through root hair ITs.

### Nodule organogenesis and rhizobial infection processes are dependent on NSP2 and NIN

In contrast to spontaneous nodulation on the roots of *nfr1-4*/CCaMK^T265D^ and *nfr5-2*/CCaMK^T265D^, no nodule structures were formed on the roots of *nsp2-1/*CCaMK^T265D^ and *nin-2/*CCaMK^T265D^ (Table 1) (Gleason *et al.*, 2006; Marsh *et al.*, 2007). Upon *M. loti* inoculation, infection defects in both *nsp2-1* and *nin-2* mutants were not restored by CCaMK^T265D^ (Table 1). The root hair phenotype of *nsp2-1/*CCaMK^T265D^ was the same as that of *nsp2-1*/wt-CCaMK, i.e. almost no micro-colonies and no ITs were formed (Heckmann *et al.*, 2006; Murakami *et al.*, 2006). Both *nin-2*/CCaMK^T265D^ (Figure 4f) and *nin-2*/wt-CCaMK showed the *nin* infection phenotypes, with abnormally curled root hairs without ITs (Schauser *et al.*, 1999). Taken together, we conclude that NSP2 and NIN both act downstream of CCaMK in both the infection process and nodule organogenesis.

### CK signaling through LHK1 is required for nodule organogenesis, but is dispensable for rhizobial infection

In addition to CCaMK, the gain-of-function LHK1 (LHK1^L266F^) also has an ability to induce spontaneous nodulation (Tirichine *et al.*, 2007). Introduction of the LHK1^L266F^ construct into several symbiotic mutants revealed that LHK1^L266F^ is epistatic to the symbiotic genes except for *NIN* and *NSP2* in nodule organogenesis (Tirichine *et al.*, 2007). To examine the involvement of LHK1 in the rhizobial infection process, LHK1^L266F^ and LHK1^L266L^ (wt-LHK1) under the control of its own promoter was introduced into symbiotic mutants. wt-LHK1 restored the infection defective phenotype of *hit1*, a loss-of-function mutant of LHK1 (Murray *et al.*, 2007). In the roots of *hit1-1*/wt-LHK1, nodule organogenesis was accompanied by infection of rhizobia, resulting in formation of fully effective nodules (Figures S3b and S4b). In contrast, both empty and effective nodules were formed on the roots of *hit1-1*/LHK1^L266F^ (Figures S3a and S4a), suggesting that LHK1^L266F^ enables the restoration of infection defects in the *hit1* mutants, while it also gives rise to a defect in the cooperative regulation of the symbiotic programs between epidermis and cortex, leading to the formation of empty nodules with no associated rhizobial infection.

In accordance with the results reported by Tirichine *et al.* (2007), spontaneous nodulation was induced on the roots of *nfr1-4*, *symrk-7*, *castor-4*, *nup85-3*, *ccamk-3* and *cyclops-4* transformed with LHK1^L266F^, indicating that LHK1^L266F^ is epistatic, in regard to nodule organogenesis, to the genes noted above (Figure S3h and Table S1). Coincidently, no spontaneous nodulation occurred on the *hit1-1*/CCaMK^T265D^ roots (Figure S3g). In contrast to nodule organogenesis, the defect in rhizobial infection of *ccamk-3* was not restored by LHK^L266F^ (Figure S3f). Moreover, LHK1^L266F^ could not restore infection defective phenotypes of the upstream mutants, *symrk-7*, *castor-4* and *nup85-3* (Figure S3d,e and Table S1). Only empty nodules were found on those roots of upstream mutants/LHK1^L266F^, as well as *nfr1-4*/LHK1^L266F^ roots (Figure S3c). These results demonstrate that the rhizobial infection process is independent of LHK1. The *hit1* mutant showed distinct symbiotic defective phenotypes: the formation of an excessive numbers of ITs in the epidermis, while IT development was arrested at the cortex (Murray *et al.*, 2007). Because of this, we also examined the effects of CCaMK^T265D^ in the rhizobial infection processes of the *hit1* mutant. On both *hit1-1*/CCaMK^T265D^ and *hit1-1*/wt-CCaMK roots, abundant ITs, majority of which did not penetrate to the cortical layer, were observed (Figure S4c,d). Very occasionally, aberrantly-developed infected nodules were formed on both mutant roots (Table 1 and Figure S4e,f), similar to the results described by Murray *et al.* (2006), who reported that the *hit1-1* mutant formed effective but irregularly shaped nodules on rare occasions. These data support the conclusion that CCaMK^T265D^ does not modulate abnormal infection phenotypes of *hit1-1*, as well as wt-CCaMK. Collectively, our results suggest that symbiotic defective phenotype of *hit1* is caused by decoupling of the infection events in the epidermis with nodule organogenesis, which is initiated in cortex (Figure S4c,d). In either rhizobial infection or nodule organogenesis, NSP2 and NIN are also positioned downstream of LHK1, because LHK1^L266F^ constructs could not suppress the defects of nodule organogenesis in *nsp2-1* and *nin-2* mutants (Table S1).

**Figure 5:**
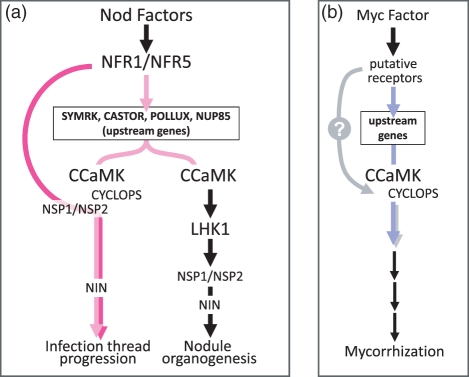
A model for regulation pathways responsible for RN and AM symbioses. (a) In response to Nod factors, the signal generated by NFR1/NFR5 splits into two pathways, one of these flows into the common symbiosis pathway (pink line). The input of another pathway (deep pink line) is prerequisite for successful infection of rhizobia. Epistasis between CYCLOPS and NSP2 on the pathway remains unclear. ITs were rarely, but initiated in *cyclops* (Yano *et al.*, 2006, 2008), while no micro-colonies were observed in *nsp2* (Murakami *et al.*, 2006). Therefore, CYCLOPS appears to be downstream of NSP2 on the pathway leading to IT formation. One possible explanation is that NSP2 may be positioned on another pathway that originates from NFR1/NFR5. For nodule organogenesis, only one signal is sufficient for activation of the downstream pathway, in which LHK1, NSP1/2 and NIN are involved. CYCLOPS is not involved in nodule organogenesis. (b) In the AM symbiosis, a plausible AM pathway that bifurcates after putative receptors (grey arrow) might be converged with common symbiosis pathway (blue arrows).

Epistatic analysis shown here supports the idea that LHK1-dependent CK signaling is positioned downstream of CCaMK in nodule organogenesis (Murray *et al.*, 2007; Tirichine *et al.*, 2007). In addition, our studies provide evidence that LHK^L266F^ could not obviate the requirement of upstream genes for rhizobial infection, indicating that rhizobial infection process is regulated by an LHK1-independent pathway. Taken together with the results of CCaMK^T265D^, we conclude that rhizobial infection process is regulated by cooperation of CCaMK and CYCLOPS, while both CCaMK and LHK1 are responsible for regulation of nodule organogenesis and both symbiotic processes are dependent on NSP2 and NIN (Figure 5a).

## Discussion

To accommodate their microsymbiotic partners properly, host plants have developed complex and highly organized signaling pathways, which perceive and process information from the symbionts and/or its own plant cell status, such as Ca^2+^ and CK signaling (Crespi and Frugier, 2008; Kosuta *et al.*, 2008; Oldroyd and Downie, 2008; Parniske, 2008). In the present work, we investigated epistatic relationships of genes involved in the early symbiotic signaling pathways, by means of transformation of gene mutants with gain-of-function CCaMK^T265D^ and LHK1^L266F^.

In the roots of the upstream gene mutants, introduction of CCaMK^T265D^ allowed rhizobia to enter host plants through ITs as well as the initiation of nodule organogenesis and thus fully compensated for the gene mutant symbiotic defects (Figure 1c–h). Similarly, CCaMK^T265D^ transformation resulted in suppression of the defects in AM symbiosis in these mutants (Figure 2b–e). These results provide conclusive evidence that the common symbiosis genes upstream of Ca^2+^ spiking are only required for the activation of CCaMK and its activation allows symbiotic interaction with rhizobia and AM fungi in *L. japonicus.* Ca^2+^ spiking is very likely to participate in activation of CCaMK, because upstream genes are essential for the generation of Ca^2+^ spiking (Miwa *et al.*, 2006); there is already an analogy to mammal CaMKII, namely the autophosphorylation of CaMKII is sensitive to the frequency of Ca^2+^ spikes (Hudmon and Schulman, 2002). Our data indicate that CaMV35S-driven CCaMK^T265D^ efficiently mimics the CCaMK that is activated in response to Ca^2+^ derived from the cytoplasm (Figures 1a,b and 2a).

In contrast to the upstream gene mutants above described, the infection defects of the NF receptor mutants, *nfr1* and *nfr5*, were never restored by CCaMK^T265D^ in our hairy root transformation system, although it could induce nodule organogenesis in these mutants irrespective of the presence or absence of *M. loti* (Figures 4a–d and S2a,b). This finding indicates that CCaMK^T265D^ alone is sufficient for the induction of cortical cell division and successive nodule organogenesis, while intracellular accommodation of rhizobia through IT within root hairs absolutely requires NF perception by NFR1 and NFR5. Therefore, our results strongly suggest that the infection signal, elicited by Nod factor perception by NFR1/NFR5 receptors in *L. japonicus*, is split into two signaling pathways; one is through Ca^2+^ spiking and is mediated by the common symbiosis genes (Figure 5a, indicated by pink arrows) and the other is separately derived from the NF receptors (Figure 5a, indicated by the deep pink arrow). The former appears to be essential and sufficient for nodule organogenesis, but the progression of the infection process via ITs additionally requires the operation of the latter (Figure 5a).

In some cases, *M. loti* can infect *L. japonicus* independently of Nod factor perception by NFR1 and NFR5, as a gain-of-function mutation of CCaMK (*snf1*) under *nfr1*/*nfr5* background was shown to be infected by *M. loti*, even though at a very low frequency (Madsen *et al.*, 2010). This NF-independent infection is, however, not via root hair ITs and rhizobia enter the cortex intercellularly. In contrast, nodules formed on *symrk-14* (Murray *et al.*, 2006) was shown to be infected by rhizobia through a process similar to ‘crack entry’, even though there is no induction of Ca^2+^ spiking in *symrk-14* (K. Szczyglowski, personal communication, 2009). Thus, the signal input from only one of the pathways may occasionally allow aberrant infection of rhizobia, but it is never accompanied by formation of root hair ITs. These observations strengthen the idea that the integration of two signaling pathways, one through Ca^2+^ spiking and another derived from NF receptors separately from the one mediated by the common symbiosis genes, is prerequisite for rhizobial infection through root hair ITs, which serve as the main route of rhizobial entry into host cells (Figure 5a).

A similar model including two signaling pathways for nodulation and IT formation has been proposed for another model legume, *M. truncatula* (Smit *et al.*, 2007). However, the framework of NF perception in *Lotus* (NFR1/NFR5) and in *Medicago* (LYK3/NFP) appears not to be exactly the same. In *L. japonicus*, NFR1 and NFR5 are required for the generation of both Ca^2+^ influx and Ca^2+^ spiking (Miwa *et al.*, 2006). Co-transformation of NFR1 and NFR5 allows *M. truncatula* to be infected by *M. loti*, suggesting that they form a receptor complex that is responsible for specific recognition of NFs derived from *M. loti* (Radutoiu *et al.*, 2003, 2007). In *M. truncatula*, NFP, a putative ortholog of NFR5 (Arrighi *et al.*, 2006; Lohmann *et al.*, 2010), is positioned upstream of both Ca^2+^ signals (Amor *et al.*, 2003). Neither root hair swelling (Has) nor root hair deformation (Had) were observed in the roots of *nfp* mutant (Amor *et al.*, 2003), as well as in the roots of *nfr5* and *nfr1 in Lotus* (Radutoiu *et al.*, 2003). Although, LYK3 has been proposed to be an ortholog of NFR1 (Arrighi *et al.*, 2006; Lohmann *et al.*, 2010), *hcl* mutants retain the ability to induce Ca^2+^ spiking, Ca^2+^ influx, Has and Had in response to *Sinorhizobium meliloti* infection (Catoira *et al.*, 2001; Smit *et al.*, 2007; Wais *et al.*, 2000), indicating that these symbiotic responses are LYK3 independent. Phenotypic divergence between *nfr1* and *hcl* implies that the position of NFR1 and LYK3 within symbiotic signaling pathway is not identical. Because of the highly strict structural requirement of LYK3 for *S. meliloti* NFs, LYK3 is proposed to be an ‘entry receptor’ that is responsible for IT formation rather than nodule primordium initiation and to be independent of the pathway mediated by the common symbiosis genes (Smit *et al.*, 2007). While another receptor complex with a lower requirement toward NF structures is postulated as a ‘signaling receptor’, which is responsible for nodule initiation through the pathway mediated by the common symbiosis genes (Smit *et al.*, 2007). In our model for *L. japonicus*, a receptor complex putatively composed of NFR1 and NFR5 is responsible for processing two signaling pathways leading to not only nodule organogenesis, but IT formation. It should be noted, however, that *in silico* searches of genome databases of *L. japonicus* and *M. truncatula* have revealed the presence of a number of LysM receptor kinases in their genome (Arrighi *et al.*, 2006; Lohmann *et al.*, 2010). In addition, *M. loti* has been shown to produce Nod factors with diverse side-chain modifications and acyl moieties (Shibata *et al.*, 2005). Thus, although applicability of the ‘signaling/entry receptor model’ to the *Lotus* NF signaling pathway(s) is still an open question, NF signaling, from the first contact of rhizobia on root hairs to the development of ITs towards the cortex, might be mediated by complex combinations of multiple LysM receptor kinases including those other than NFR1 and NFR5. Nevertheless, our data demonstrate that NFR1 and NFR5 are both essential for initiating two signaling pathways for nodule primordium formation and IT formation.

It has recently been proposed that the symbiotic signal transduction pathway(s) is bifurcated at or just downstream of CCaMK; one is a CYCLOPS-dependent pathway required for initiation of ITs and the other is a CYCLOPS-independent pathway leading to nodule organogenesis (Yano *et al.*, 2008). In accordance with this proposal, we have shown that the CYCLOPS-dependent pathway regulates both rhizobial and AM fungal infection processes (Figure 3c–e,g,h). It has been reported that the size of spontaneous nodules formed on the roots of *cyclops-4*/CCaMK^T265D^ did not differ from those formed on wt/CCaMK^T265D^ roots under mock inoculation (Yano *et al.*, 2008). However, restoration of nodule organogenesis appeared to be impaired in response to rhizobial inoculation, i.e. nodule organogenesis on *cyclops-4*/CCaMK^T265D^ roots remained at the stage of small bumps when inoculated with *M. loti* (Figure 3c). A similar phenotype has been described for the roots of *cerberus*/CCaMK^T265D^ (Yano *et al.*, 2009). *CERBERUS* encodes a novel U-box protein containing WD-40 repeats and is shown to be essential for the development of ITs. On both *cyclops*/CCaMK^T265D^ and *cerberus*/CCaMK^T265D^ roots, bump formation were induced by *M. loti* inoculation, while spontaneous nodules with genuine nodule structure were developed under mock inoculation (Yano *et al.*, 2008, 2009). In the case of *nfr1-4*/CCaMK^T265D^ and *nfr5-2*/CCaMK^T265D^, the deficiency in the ability to recognize NFs resulted in complete loss of infection events in epidermis even in the presence of *M. loti.* Taken together, developmental arrest of ITs in the epidermis appears to affect adversely the progression of nodule organogenesis in the cortex.

In this study, we showed that the introduction of LHK1^L266F^ could not rescue the infection defective phenotypes of the upstream gene mutants, in contrast to CCaMK^T265D^. Lohar *et al.* (2004) has demonstrated that CK signaling is activated in response to NFs in *L. japonicus;* CK-responsive Arabidopsis response regulator (ARR5) promoter-GUS expression was induced along symbiosis with *M. loti*, in deformed root hairs, dividing cortical cells and nodule primordia. Although the role of CK in rhizobial infection processes remains to be proven, CK signaling through LHK1 appears to be not necessary for IT formation in the RN symbiosis. It is believed that coordinated regulation of rhizobial infection and nodule organogenesis is essential for the development of effective nodules (Frugier *et al.*, 2008; Gonzalez-Rizzo *et al.*, 2006; Murray *et al.*, 2007; Oldroyd, 2007). The phenotype of *hit1-1*/LHK1^L266F^ appeared to be due to cortical cell division which could not coupled appropriately with IT development within root hairs, thus leading to the formation of a large number of empty nodules, even though it also formed effective nodules on much rarer occasions (Figure S4a and Table S1). IT formation program functions first in the epidermis, while nodule primordium formation, which involves LHK1-mediated CK signaling, occurs in root cortical cells. Our results, together with the infection defective phenotype of *cyclops*/CCaMK^T265D^, indicate that disturbance of nodule organogenesis programs by LHK1^L266F^ adversely affects the rhizobial infection process, suggesting that coordinated regulation of symbiotic signaling cascades between epidermis and cortex is essential for the establishment of successful symbiosis.

The RN symbiosis is assumed to have evolved by recruiting the pre-existing common symbiosis genes for the AM symbiosis (Markmann and Parniske, 2009; Parniske, 2008). Indeed, non-leguminous orthologs of common symbiosis genes have been isolated and their involvement in the AM symbiosis has been proven in rice (Banba *et al.*, 2008; Chen *et al.*, 2007, 2008, 2009; Gutjahr *et al.*, 2008; Markmann *et al.*, 2008; Yano *et al.*, 2008; Zhu *et al.*, 2006). Leguminous plants have an ability to interact with rhizobia in addition to AM fungi. This means that leguminous plants can distinguish different symbionts and regulate respective pathways appropriately. Although RN and AM symbioses share common symbiosis genes that play roles in signal transduction mediated by Ca^2+^ spiking, leguminous plants can open different gates for different symbionts. It has been shown that Ca^2+^ spiking has different signatures depending on RN or AM symbiotic interactions, leading to the transmission of RN- or AM-specific information to the downstream pathways (Kosuta *et al.*, 2008). However, in the roots of the upstream gene mutants/CCaMK^T265D^, the gain-of-function status of CCaMK^T265D^ is apparently identical regardless of whether the roots are infected by rhizobia or AM fungi, implying that a specific signal(s), other than those mediated by common symbiosis genes, plays a role in determination of downstream pathways responsible for each of the symbioses. In the case of the upstream mutants/CCaMK^T265D^, the gain-of-function status of CCaMK^T265D^ driven by CaMV35S promoter is presumed to far exceed the threshold of the CCaMK activity required for intracellular infections of both rhizobia and AM fungi. During evolution of the RN symbiosis, leguminous plants were likely to have acquired not only the competence to transmit RN-specific signals through the common symbiosis genes, but also another RN-specific signaling pathway directly originated from the NF receptors. Based on cross-species complementation analyses of leguminous common symbiosis mutants with corresponding rice ortholog genes, most of the common symbiosis genes show functional conservation in AM and RN symbioses (Chen *et al.*, 2007; Banba *et al.*, 2008; Yano *et al.*, 2008), while only SYMRK has the distinctive position as the adaptive factor that confers the RN symbiosis on leguminous plants (Markmann *et al.*, 2008). It is of great interest to analyze the correlation between the domain composition of SYMRK and the signal intensity of Ca^2+^ spiking induced by respective SYMRK orthologs.

Although it remains unclear whether CCaMK^T265D^ only represents CCaMK activated by cytosolic Ca^2+^ derived from Ca^2+^ spiking, our results suggest that CCaMK^T265D^ is sufficient for intracellular rhizobial infection when it has received an input from another signaling cascade(s), which is derived directly from NFR1 and NFR5 separately from that involving Ca^2+^ spiking. Among well characterized physiological reactions of host cells in response to NF application, Ca^2+^ influx may be a good candidate for another signal derived from Nod factor perception (Geurts *et al.*, 2005; Miwa *et al.*, 2006; Shaw and Long, 2003). In fact, NFR1/NFR5 are required for induction of both Ca^2+^ influx and Ca^2+^ spiking, while the mutants of upstream genes are able to induce Ca^2+^ influx (Miwa *et al.*, 2006). Although the convergence of these two Ca^2+^ signals in the symbiotic signal transduction cascades remains to be elucidated, candidates responsible for signal integration are likely to be capable of binding Ca^2+^. Kinase-only CCaMK, which lacks both the CaM-binding domain and EF hands, induces spontaneous nodulation, while neither rhizobial colonization nor IT initiation are observed on the roots of *dmi3*/kinase-only DMI3 in *M. truncatula* (Gleason *et al.*, 2006). This finding indicates that the Ca^2+^ binding capacity of CCaMK is necessary for rhizobial infection and a possible function of CCaMK is as the acceptor of the two Ca^2+^ signals. This idea is consistent with the proposal of Miwa *et al.* (2006) that accumulation of NF caused by *M. loti* colonization within curled root hairs leads to Ca^2+^ influx, which may drive infection thread growth in *Lotus.*

Mycorrhizal infection might also require another specific signal derived from AM specific pathway (Figure 5b) in a similar way to the RN symbiosis. Indeed, it has been reported that *MtENOD11* expression in response to mycorrhizal infection is independent of DMI genes (Kosuta *et al.*, 2003). These results suggest the presence of an AM specific pathway which might determine downstream AM specific pathway(s) to be activated (Figure 5b).

The function of symbiotic genes to different cellular responses, leading to rhizobial infection and to nodule organogenesis, is a complex biological problem. To further elucidate the regulation pathways responsible for RN symbiosis, we focused on evaluating the possible involvement of a number of symbiotic genes in the infection process, by analyzing the infection phenotypes of corresponding mutants with expression of gain-of-function CCaMK and LHK1. Taken together with epistatic analyses on the basis of spontaneous nodulation and infection phenotypes, we demonstrate that the compositions of gene sets responsible for the infection process (IT formation) and nodule organogenesis are not equal, even though they share the same components in part (Figure 5a). In addition, our results clearly indicate the key role(s) of CCaMK in both rhizobial infection and the nodule organogenesis program.

The common symbiosis genes are considered to be a conserved genetic pathway for the AM symbiosis and to act as a generator of symbiotic signals, i.e. Ca^2+^ spiking, in response to rhizobia and AM fungi interactions (Kosuta *et al.*, 2008; Markmann and Parniske, 2009). We propose that activation of CCaMK through the common symbiosis genes confers competence for the accommodation of rhizobia or AM fungi intracellularly. The study presented herein reveals dominant roles for CCaMK in endosymbioses and also raises the question of how CCaMK is activated differently by bacterial and fungal symbionts in leguminous plants.

## Experimental procedures

### Biological materials

Detailed information of *L. japonicus* used in this study is provided in Data S1. To visualize the infection processes of rhizobia, *M. loti* MAFF303099 constitutively expressing DsRed (Maekawa *et al.*, 2009) or MAFF303099 derivative ML001 constitutively expressing the β-galactosidase (lacZ) (Tansengco *et al.*, 2003) were inoculated onto hairy roots of *L. japonicus.* To examine mycorrhization phenotype, *Glomus intraradices* DAOM 197198 (Premier Tech, http://www.premiertech.com/) was used (Banba *et al.*, 2008).

### Plasmid construction

Detailed information is provided in Data S1.

### Transformation of CCaMK and LHK1 constructs

wt-CCaMK, CCaMK^T265D^, LHK1 and LHK1^L266F^ constructs were introduced into the *L. japonicus* mutants by hairy root transformation with *Agrobacterium rhizogenes*LBA1334 as described previously (Maeda *et al.*, 2006). Plants with GFP-positive hairy roots were selected by GFP fluorescence using a Leica MZFLIII stereomicroscope (Leica, http://www.leica-microsystems.com/).

### Examination of spontaneously nodulated plants

To examine the extent of spontaneous nodulation, transformants were transplanted into vermiculite pots supplied with B&D medium supplemented with 0.5 μm ammonium nitrate (Banba *et al.*, 2008). Four weeks after transplantation, GFP-positive roots were selected again and the spontaneous nodulation phenotype was observed with a Leica MZFLIII stereomicroscope.

### Inoculation tests with rhizobial or mycorrhizal strains

For characterization of infection phenotypes, transformants were transplanted into vermiculite pots supplied with B&D medium supplemented with 0.5 μm ammonium nitrate. Three days after transplantation, *M. loti* strains were inoculated. For mycorrhizal inoculation, transformants were transplanted into an autoclaved 1:1 mixture of lawn soil (Shibametsuchi) and a nutrient-rich commercial horticulture soil (Kureha, http://www.kureha.co.jp/), as described previously (Banba *et al.*, 2008). *Glomus intraradices* was inoculated using approx. 200 spores per plant. The plants were grown in a growth cabinet with a 16 h-day/8 h-night cycle at 24°C. Four weeks after inoculation, plants with GFP-positive hairy roots were selected using a Leica MZFLIII stereomicroscope.

### Histological examination of rhizobial infection phenotypes

To examine the extent of rhizobial infection and nodule organogenesis, lacZ-expressing *M. loti* was visualized with a chemical staining method as described previously (Tansengco *et al.*, 2003) and observed using a Leica MZFLIII stereomicroscope. DsRed-expressing *M. loti* was observed using a 565/595 nm bandpass filter and a CCD camera system (Penguin 600CL; Pixera, http://www.pixera.com/) attached to the Leica MZFLIII stereomicroscope. For observation of ITs, samples were analyzed under an epifluorescence microscope (BZ-9000; Keyence, http://www.keyence.co.jp/) using a filter set (excitation BP560–600, dichroic 595, emission BP630–690).

### Histological observations of mycorrhization phenotypes

*Glomus intraradices*-inoculated roots were stained with trypan blue as described previously (Saito *et al.*, 2007; Banba *et al.*, 2008). Hyphal or arbuscule colonization was observed under a bright-field microscope (Leitz DMRB, Leica) with a CCD camera system (Penguin 600CL; Pixera).

## SUPPORTING INFORMATION

**Data S1.** Experimental procedures.

**Figure S1.** Transformation of wt-CCaMK or CCaMKT^265D^ in the *ccamk-3* mutant.

**Figure S2.** Transformation of CCaMKT^265D^ in non-nodulating mutants.

**Figure S3.** Complementation of rhizobial infection phenotypes of non-nodulating mutants by LHK1^L266F^ transformation.

**Figure S4.** Transformation of LHK1^L266F^, wt-LHK1, CCaMK^T265D^ or wt-CCaMK in *hit1-1* mutant.

**Table S1.** Induction of spontaneous nodulation and restoration of symbiotic defective phenotypes of non-nodulating mutants, transformed with LHK1^L266L^ (LHK1) or gain of function LHK1^L266F^ (gof- LHK1) constructs.

Please note: As a service to our authors and readers, this journal provides supporting information supplied by the authors. Such materials are peer-reviewed and may be re-organized for online delivery, but are not copy-edited or typeset. Technical support issues arising from supporting information (other than missing files) should be addressed to the authors.
