# Supplementary material for: A dominant function of CCaMK in intracellular accommodation of bacterial and fungal endosymbionts
Source: Plant J. 2010 May 11;63(1):141–54. doi: 10.1111/j.1365-313X.2010.04228.x (PMC2916219; doi:10.1111/j.1365-313X.2010.04228.x)
Supplement: Supplementary file 1 [file tpj0063-0141-SD1.doc]

**Experimental procedures S1**

**Detailed information of *L. japonicus* used in this study**

*L. japonicus* ecotype Gifu WT and *nfr1-4* (Sandal *et al.*, 2006), *nfr5-2* (Madsen *et al.*, 2003), *symrk-3* (Stracke *et al.*, 2002; Kistner *et al.*, 2005), *symrk-7* (Stracke *et al.*, 2002; Kistner *et al.*, 2005), *castor-4* (Imaizumi-Anraku *et al.*, 2005), *pollux-2* (Imaizumi-Anraku *et al.*, 2005), *nup85-3* (Saito *et al.*, 2007), *ccamk-3* (Tirichine *et al.*, 2006), *cyclops-4* (Yano *et al.*, 2008), *nsp2-1* (Murakami *et al.*, 2006), *nin-2* (Schauser *et al.*, 1999) and *hit1-1* (Murray *et al.*, 2007) mutants were used in this work.

**Detailed information of plasmid construction in this study**

The procedure for construction of the gain-of-function CCaMK^T265D^ was described previously (Yano *et al.*, 2008). For the construction of LHK1, the genomic LHK1 region was amplified by PCR with AscI-LHK1-f: GGCGCGCCAGTCTGATCATTGTCTCAATTCCA/LHK1-AscI-r: GGCGCGCCATGAAATTCTTCTCCAAGGAAGTG primer pair. The fragment was digested with *Asc*I and ligated with p35S-GFP vector (Yano *et al.*, 2008). The gain-of-function LHK1^L266F^ (Tirichine *et al.*, 2007) was constructed as follows: the mutation was induced by PCR with two primer pairs, AscI-LHK1-f/LHK1-L266F-r: GCTTGGTTACCAGCAAGTTGACCAAATAAA and LHK1-L266F-f:AGTCCCTTGTGGAGAATTTATTTG/LHK1-AscI-r. Respective PCR fragments were digested with *Asc*I and *Bst*EII, and then ligated with p35S-GFP vector.

**References**

Sandal N, Petersen TR, Murray J, Umehara Y, Karas B, Yano K, Kumagai H, Yoshikawa M, Saito K, Hayashi M, Murakami Y, Wang X, Hakoyama T, Imaizumi-Anraku H, Sato S, Kato T, Chen W, Hossain MS, Shibata S, Wang TL *et al.* (2006) Genetics of symbiosis in *Lotus japonicus*: recombinant inbred lines, comparative genetic maps, and map position of 35 symbiotic loci. *Mol Plant Microbe Interact* 19: 80–91.
