# Supplementary figures and images for: A dominant function of CCaMK in intracellular accommodation of bacterial and fungal endosymbionts
Source: Plant J. 2010 May 11;63(1):141–54. doi: 10.1111/j.1365-313X.2010.04228.x (PMC2916219; doi:10.1111/j.1365-313X.2010.04228.x)

Figure S1

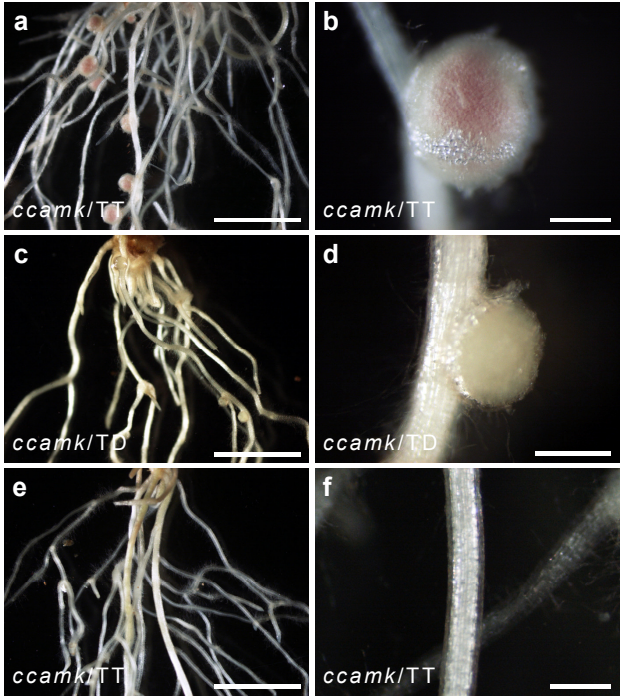

Figure S2

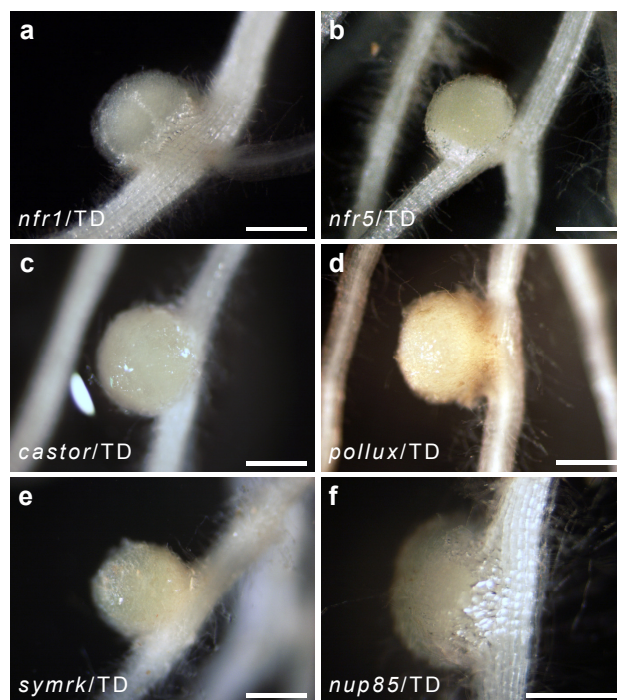

Figure S3

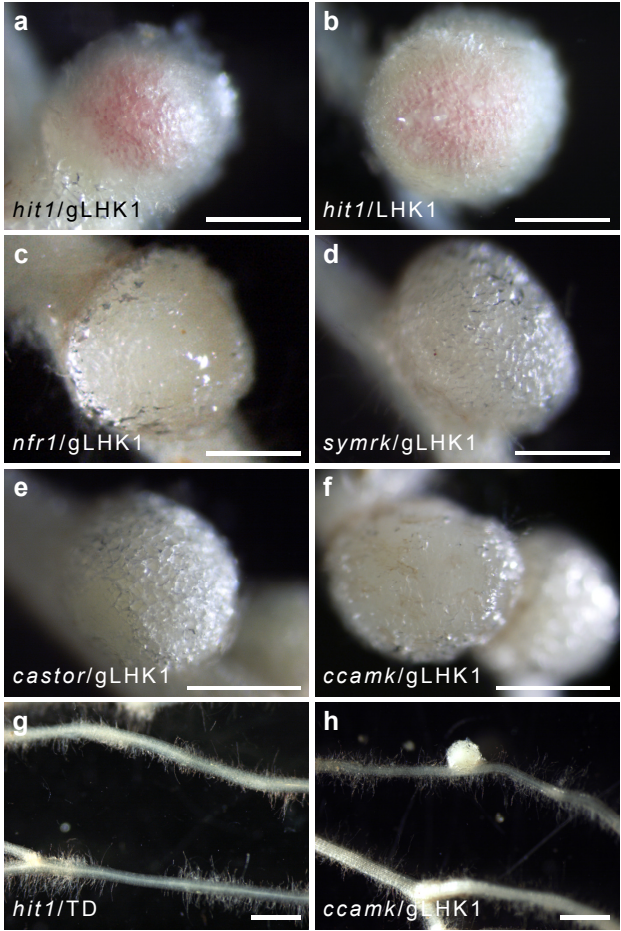

Figure S4

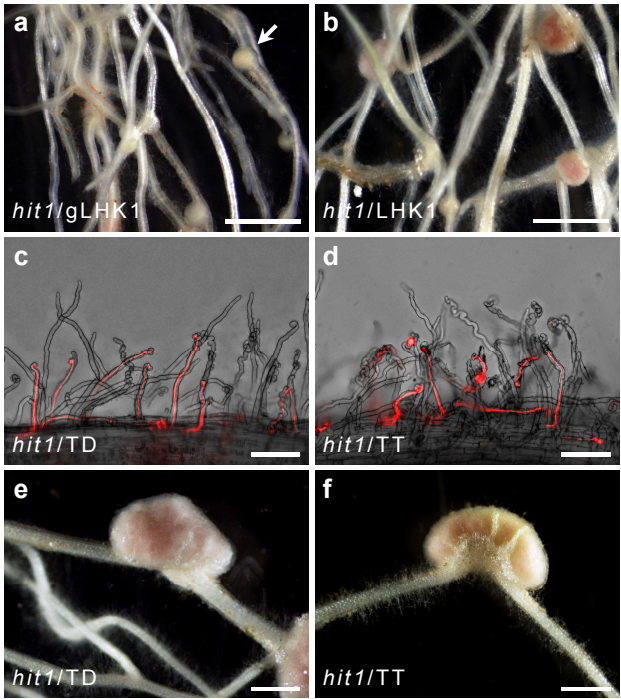

Supplement: Supplementary file 2 [file tpj0063-0141-SD2.pdf]
