# Supplementary material for: A dominant function of CCaMK in intracellular accommodation of bacterial and fungal endosymbionts
Source: Plant J. 2010 May 11;63(1):141–54. doi: 10.1111/j.1365-313X.2010.04228.x (PMC2916219; doi:10.1111/j.1365-313X.2010.04228.x)
Supplement: Supplementary file 3 [file tpj0063-0141-SD3.doc]

| Table S1. Induction of spontaneous nodulation and restoration of symbiotic defective phenotypes of non-nodulating mutants, transformed with LHK1^L266L^ (LHK1) or gain of function LHK1^L266F^ (gof-LHK1) constructs. | | | |
| --- | --- | --- | --- |
| *Lotus* lines | LHK1 construct | phenotypes | |
|  |  | SpN^a^ | Nod^b^ |
| Gifu (B-129) | LHK1 | 0/30 | 12/12 |
| Gifu (B-129) | gof-LHK1 | 29/72 | 22/22 |
| *hit1-1* | LHK1 | 0/17 | 9/11 |
| *hit1-1* | gof-LHK1 | 13/20 | 4/4 |
| *nfr1-4* | LHK1 | 0/11 | 0/13 |
| *nfr1-4* | gof-LHK1 | 5/15 | 19/41^c^ |
| *symrk-7* | LHK1 | 0/8 | 0/9 |
| *symrk-7* | gof-LHK1 | 17/35 | 11/31^c^ |
| *castor-4* | LHK1 | 0/10 | 0/10 |
| *castor-4* | gof-LHK1 | 6/16 | 11/34^c^ |
| *nup85-3* | LHK1 | 0/4 | 0/12 |
| *nup85-3* | gof-LHK1 | 6/14 | 14/24^c^ |
| *ccamk-3* | LHK1 | 0/18 | 0/17 |
| *ccamk-3* | gof-LHK1 | 6/56 | 2/45^c^ |
| *cyclops-4* | LHK1 | 0/25 | 35/35^d^ |
| *cyclops-4* | gof-LHK1 | 10/22 | 22/22^d^ |
| *nsp2-1* | LHK1 | 0/8 | 0/11 |
| *nsp2-1* | gof-LHK1 | 0/8 | 0/12 |
| *nin-2* | LHK1 | 0/5 | 0/13 |
| *nin-2* | gof-LHK1 | 0/20 | 0/16 |
| a: spontaneous nodulation in the absence of *M. loti*, b: nodule formation under *M. loti* inoculation, c: empty nodules without rhizobial invasion, d:bump-like structure without rhizobial invasion. Number of plants with the phenotypes described above (a~d) per number of transformed plants are listed. Phenotypes were examined 6 weeks after transplantation or *M. loti* inoculation. | | | |
